# Kidney and vascular function in adult patients with hereditary fructose intolerance

**DOI:** 10.1016/j.ymgmr.2020.100600

**Published:** 2020-05-11

**Authors:** Nynke Simons, François-Guillaume Debray, Nicolaas C. Schaper, Edith J.M. Feskens, Carla E.M. Hollak, Judith A.P. Bons, Jörgen Bierau, Alfons J.H.M. Houben, Casper G. Schalkwijk, Coen D.A. Stehouwer, David Cassiman, Martijn C.G.J. Brouwers

**Affiliations:** a Division of Endocrinology, Department of Internal Medicine, Maastricht University Medical Center, Maastricht, The Netherlands; b Laboratory for Metabolism and Vascular Medicine, Division of General Internal Medicine, Department of Internal Medicine, Maastricht University Medical Center, Maastricht, The Netherlands; c CARIM School for Cardiovascular Diseases, Maastricht, The Netherlands; d Department of Medical Genetics, Metabolic Unit, University Hospital Liège, Liège, Belgium; e CAPHRI School for Public Health and Primary Care, Maastricht, The Netherlands; f Division of Human Nutrition, Wageningen University, Wageningen, The Netherlands; g Division of Endocrinology and Metabolism, Department of Internal Medicine, Academic Medical Center, Amsterdam, The Netherlands; h Central Diagnostic Laboratory, Maastricht University Medical Center, Maastricht, The Netherlands; i Department of Clinical Genetics, Maastricht University Medical Center, Maastricht, The Netherlands; j Division of General Internal Medicine, Department of Internal Medicine, Maastricht University Medical Center, Maastricht, The Netherlands; k Department of Gastroenterology-Hepatology and Metabolic Center, University Hospital Leuven, Leuven, Belgium

**Keywords:** Case-control study, Hereditary fructose intolerance, Fanconi syndrome, Kidney, Blood, Vessels, 95% confidence interval, (95% CI), alanine, (Ala), aldolase B, (ALDOB), arginine, (Arg), asparagine, (Asn), carotid-femoral pulse wave velocity, (cf-PWV), chronic kidney disease epidemiology collaboration, (CKD-EPI), CKD-EPI equation based on serum creatinine, (eGFRcr), CKD-EPI equation based on cystatin c, (eGFRcys), CKD-EPI equation based on creatinine and cystatin c combined, (eGFRcr-cys), citrulline, (Cit), cysteine, (Cys), difference, (Δ), estimated glomerular filtration rate, (eGFR), soluble E-selectin, (sE-selectin), glucokinase regulatory protein, (GKRP), glutamine, (Gln), glutamic acid, (Glu), glycine, (Gly), hereditary fructose intolerance, (HFI), histidine, (His), intrahepatic triglyceride, (IHTG), isoleucine, (Ile), leucine, (Leu), lysine, (Lys), methionine, (Met), ornithine, (Orn), perfusion units, (PU), reactive hyperemia peripheral arterial tonometry, (RH-PAT), phenylalanine, (Phe), proline, (Pro), ratio of tubular maximum reabsorption of phosphate to GFR, (TmP/GFR), reactive hyperemia index, (RHI), serine, (Ser), laser doppler flowmetry, (LDF), statistical package of social sciences, (SPSS), taurine, (Tau), threonine, (Thr), tryptophan, (Try), tubular reabsorption of phosphate, (TRP), tyrosine, (Tyr), valine, (Val), von willebrand factor, (vWF)

## Abstract

*Objective*: Previous studies have shown that patients with hereditary fructose intolerance (HFI) are characterized by a greater intrahepatic triglyceride content, despite a fructose-restricted diet. The present study aimed to examine the long-term consequences of HFI on other aldolase-B-expressing organs, i.e. the kidney and vascular endothelium.

*Methods*: Fifteen adult HFI patients were compared to healthy control individuals matched for age, sex and body mass index. Aortic stiffness was assessed by carotid-femoral pulse wave velocity (cf-PWV) and endothelial function by peripheral arterial tonometry, skin laser doppler flowmetry and the endothelial function biomarkers soluble *E*-selectin [sE-selectin] and von Willebrand factor. Serum creatinine and cystatin C were measured to estimate the glomerular filtration rate (eGFR). Urinary glucose and amino acid excretion and the ratio of tubular maximum reabsorption of phosphate to GFR (TmP/GFR) were determined as measures of proximal tubular function. *Results*: Median systolic blood pressure was significantly higher in HFI patients (127 versus 122 mmHg, *p* = .045). Pulse pressure and cf-PWV did not differ between the groups (*p* = .37 and *p* = .49, respectively). Of all endothelial function markers, only sE-selectin was significantly higher in HFI patients (*p* = .004). eGFR was significantly higher in HFI patients than healthy controls (119 versus 104 ml/min/1.73m^2^, *p* = .001, respectively). All measurements of proximal tubular function did not differ significantly between the groups.

*Conclusions*: Adult HFI patients treated with a fructose-restricted diet are characterized by a higher sE-selectin level and slightly higher systolic blood pressure, which in time could contribute to a greater cardiovascular risk. The exact cause and, hence, clinical consequences of the higher eGFR in HFI patients, deserves further study.

## 1 Introduction

Hereditary fructose intolerance (HFI; OMIM# 229600) is an autosomal recessive metabolic disorder [1] with an estimated incidence of 1 in every 20,000 newborns [2]. HFI is caused by mutations in the gene encoding aldolase B (*ALDOB*) that is predominantly expressed in liver, kidney and small intestine [3]. Aldolase B (EC 4.1.2.13) catalyzes the conversion of fructose 1,6-bisphosphate and fructose 1-phosphate to glyceraldehyde (3-phosphate) and dihydroxyacetone phosphate [4]. Fructose ingestion in HFI patients causes accumulation of fructose 1-phosphate, and intracellular phosphate and ATP depletion, resulting in acute and chronic cellular dysfunction. In the short term, it produces nausea, vomiting, abdominal pain and hypoglycemia. Chronic exposure to fructose can result in cirrhosis, liver failure, generalized proximal tubular dysfunction (i.e. Fanconi syndrome), growth retardation or even death [[5], [6], [7], [8], [9]].

Treatment of HFI consists of adherence to a diet that is devoid of fructose [10]. Although this diet is adequate in providing a relatively normal, symptom-free life, little is known about the long-term consequences of HFI and its related diet.

We and others recently showed that HFI patients treated with a fructose-restricted diet are characterized by more liver fat [[11], [12], [13]] and glucose intolerance compared to healthy controls [11]. In addition, one out of 15 HFI patients had clinically relevant liver fibrosis [11].

To date, the long-term effects of HFI on other aldolase-B-expressing organs, such as the kidney, have not been described. Experimental studies have shown that aldolase B is also expressed in endothelial cells [14], vascular smooth muscle cells and aorta [15,16], and is actively involved in vascular remodeling [15].

Therefore, the aim of the present study was to investigate blood pressure, aortic stiffness, endothelial function, glomerular function and proximal tubular function in adult HFI patients treated with a fructose-restricted diet.

## 2 Materials and methods

### 2.1 Study population

Details of this study have recently been described [11]. Briefly, in this case-control study patients with a confirmed diagnosis of HFI (either via a fructose tolerance test, measurement of aldolase B activity in liver biopsy tissue or DNA analysis) were recruited from several outpatient metabolic clinics in the Netherlands and Belgium and compared to healthy control individuals matched for age, sex and BMI. Exclusion criteria for participation were contra-indications for magnetic resonance imaging (the quantification of intrahepatic fat was the primary outcome measurement of this study [11]) or inability to give informed consent. Participants visited the metabolic ward after an overnight fast of at least eight hours. All measurements were performed in the fasting state. All participants gave written informed consent prior to inclusion in the study. The study was performed according to the Declaration of Helsinki [17] and approved by the medical ethical committee of Maastricht University Medical Center.

### 2.2 Measurements of outcomes

#### 2.2.1 Blood pressure, pulse wave velocity and endothelial function

Measurement of systolic and diastolic blood pressure was done twice in sitting position on the right upper arm after 10 min of rest with 3 min interval (Omron, Hoofddorp, The Netherlands). The mean of the two measurements was used for further analysis. Pulse pressure was calculated as the difference between systolic and diastolic blood pressure.

Aortic stiffness – i.e. the cushioning capacity of the aorta, which is a determinant of systolic and diastolic blood pressure and, hence, a cardiovascular risk factor [18] – was estimated by carotid-femoral pulse wave velocity (cf-PWV) using the SphygmoCor device (AtCor Medical, Sydney, Australia), which is based on the speed of travel of a pulse along an arterial segment [19]. All measurements were performed in the supine position after 10 min of rest. The median of at least three adequate measurements was used for statistical analysis.

The function of the endothelium – the vascular barrier that regulates the availability of nitric oxide (and, hence, vascular tone), permeability, leukocyte adhesion, and procoagulant activity [20] – was assessed by several function tests (peripheral arterial tonometry and skin laser doppler flowmetry) and serum biomarkers (soluble *E*-selectin and von Willebrand). First, we performed peripheral arterial tonometry (RH-PAT; ENDOPAT Itamar Medical, Caesarea, Israel), which measures digital pulsatile arterial pressure changes accompanying pulse waves during reactive hyperemia after local ischemia [21]. For this, a blood pressure cuff was placed on the right upper arm and a RH-PAT probe, consisting of two finger-mounted probes, was placed on one finger (digitus III) of both the right (test) and left (control) hand. After an equilibration period of 5 min, the blood pressure cuff was inflated to a suprasystolic pressure for 5 min. The cuff was subsequently deflated and RH-PAT recording was proceeded for another 5 min. The reactive hyperemia index (RHI) was calculated as the ratio of the relative change in PAT signal amplitude in the test arm (= the average PAT signal amplitude during reactive hyperemia divided by the average PAT signal amplitude during baseline) over the control arm. Lower RHI indicates a diminished post-occlusion hyperemia, which reflects endothelial dysfunction of the small arteries. Second, we performed skin laser doppler flowmetry (LDF) using a laser-Doppler system (Periflux 5000, Perimed, Järfalla, Sweden) accoutered with a thermostatic laser-Doppler probe (PF457; Perimed), which measures changes in microcirculatory blood flow on the dorsal side of the left lower arm during local heating, using changes in the wavelength imparted by moving blood cells to the probing light [22]. All measurements were performed in a climate-controlled room at 24 °C [23]. The percentage increase of the skin blood flow (measured in perfusion units (PU)) during local heating over the baseline skin blood flow was used to quantify the heat-induced skin hyperemic response. Lower heat-induced skin hyperemia reflects microvascular endothelial dysfunction [24].

In addition to RH-PAT and LDF, plasma soluble *E*-selectin (sE-selectin; an endothelium-specific marker that facilitates leucocyte adhesion) and von Willebrand factor (vWF; an endothelial marker that regulates coagulation) were measured using a Diaclone ELISA kit (Diaclone SAS, Besançon Cedex, France) and Sandwich ELISA with commercially available antibodies (i.e. A0082 and P0226; Agilent DAKO, Santa Clara, CA, US).

#### 2.2.2 Glomerular and proximal tubular function

Serum cystatin C (Particle-enhanced nephelometric immunoassay, Behring Nephelometer II, Siemens Healthineers AG, Munich, Germany) and creatinine (Enzymatic colorimetric assay, Cobas 8000 instrument, Roche Diagnostics, Mannheim, Germany) were determined for the calculation of the eGFR, using the Chronic Kidney Disease Epidemiology Collaboration (CKD-EPI) Eq. [25], based on serum creatinine (eGFR_cr_), cystatin C (eGFR_cys_) and creatinine and cystatin C combined (eGFR_cr-cys_). The latter was used as a primary outcome measurement. Proteinuria was determined by measuring the total protein concentration in twenty-four-hour urine samples collected in pre-acidified plastic containers (Turbidimetric assay, Cobas 8000 instrument, Roche Diagnostics, Mannheim, Germany).

Proximal tubular dysfunction was determined by measuring the concentration of phosphate (Colorimetric assay, Cobas 8000 instrument, Roche Diagnostics, Mannheim, Germany), glucose (d-Glucose/d-Fructose UV-method, R-Biopharm AG [produced by Roche Diagnostics, Mannheim, Germany]) and amino acid (ultra-performance liquid chromatography tandem mass spectrometry, Waters, Milford, MA, US [26]) in the same twenty-four hour urine samples. To control for influential variables (e.g. diet and hormones), the tubular reabsorption of phosphate (TRP) and subsequently ratio of tubular maximum reabsorption to GFR (TmP/GFR) was calculated [27].

### 2.3 Measurements of covariates

Dietary intake was evaluated using a three-day food journal along with a personal interview by the clinical researcher (N.S.). The weight of each consumed product was either provided by the participant or estimated using average quantities per portion. Macro- and micronutrient composition was determined based on the Dutch food composition table [28]. Fructose intake was calculated with an extensive sugar composition table, as described previously [11,29].

Height was determined standing upright against a stadiometer and weight was assessed in solely underwear using an electronic scale. BMI was calculated as weight (kg) divided by height (m) squared. Waist was measured with a measuring tape at the level of the umbilicus.

Blood was drawn for measurement of whole blood glucose (YSI, Yellow Springs, OH, USA), plasma insulin (Human insulin kit, Meso Scale Discovery, Rockville, MD, USA), and serum lipids (Enzymatic colorimetric assay, Cobas 8000 instrument, Roche Diagnostics, Mannheim, Germany).

Urinary sodium concentration (Indirect ISE-method, Cobas 8000 instrument, Roche Diagnostics, Mannheim, Germany) was determined in the twenty-four-hour urine samples.

### 2.4 Statistical analyses

Data are expressed as median (interquartile range) and analyzed with a Mann-Whitney *U* test.

Blood pressure, cf-PWV, endothelial function (reactive hyperemia index, heat-induced skin hyperemia, plasma sE-selectin and vWF concentration), glomerular function (eGFR_cr-cys_ and urinary total protein concentration) and proximal tubular function (TmP/GFR, urinary glucose and amino acid concentrations) were considered as the primary outcome measurements.

The 95% confidence interval (95% CI) for the difference (Δ) between medians of HFI patients versus healthy controls as part of the Mann-Whitney test was calculated according to the Hodges-Lehmann method.

Linear regression analyses were conducted to explore the contribution of potential mediators of any difference in the primary outcome measures. A ≥ 25% reduction in the unstandardized beta of the primary outcome measurement after inclusion of the potential mediator was considered relevant. Due to the low number of study participants (*n* = 30), linear regression was conducted with only one potential mediator per analysis.

Results were considered statistically significant at *p* < .05. All analyses were carried out with the IBM Statistical Package of Social Sciences (SPSS) version 23 for Windows (SPSS inc. Chicago, IL).

## 3 Results

### 3.1 General characteristics

Fifteen HFI patients and fifteen age-, sex- and BMI-matched healthy controls were included in this study. General characteristics are displayed in Table 1, as published previously [11]. HFI patients were relatively young (median age: 31 years), lean (median BMI: 20.4 kg/m^2^) and predominantly male (73.3%). Three out of 15 HFI patients were smokers, as opposed to none of the healthy controls. Alcohol intake was higher among healthy controls (median units per day: 0.6 versus 0.1, *p* = .04). As expected, patients consumed almost no fructose (median intake: 1.0 g/day) and, as a consequence, protein and saturated fat intake were higher compared to healthy controls (median intake: 113.1 g/day versus 75.4 g/day, *p* = .005; and 37.2 g/day versus 30.0 g/day, *p* = .03, respectively). Serum lipids, whole blood glucose and plasma insulin levels did not differ significantly between both groups. None of the participants used blood pressure-, lipid- or glucose-lowering medication.

**Table 1:** General characteristics.

	Healthy controls	HFI patients
Sex (M/F)	11/4	11/4
Age (y)	28 (25–52)	31 (24–44)
BMI (kg/m^2^)	21.8 (21.0–23.3)	20.4 (19.3–24.8)
Waist circumference (cm)	87 (84.3–91.3)	77.5 (73.8–93.5)
IHTG content (%)	0.59 (0.29–1.65)	2.47 (0.76–4.56)*
Liver stiffness (kPa)	4.3 (3.6–5.5)	4.7 (4.1–6.2)
Whole blood glucose (mmol/L)	4.4 (4.2–4.7)	4.4 (4.3–4.8)
Insulin (pg/ml)	105 (77–159)	115 (93–175)
Total cholesterol (mmol/l)	4.1 (3.9–5.2)	5.0 (4.3–6.2)
HDL cholesterol (mmol/l)	1.4 (1.1–1.8)	1.6 (1.4–2.3)
LDL cholesterol (mmol/l)	2.4 (1.8–3.3)	3.1 (2.0–4.2)
Triglycerides (mmol/l)	0.9 (0.5–1.2)	0.8 (0.7–1.3)
Uric acid (μmol/l)	334 (285–378)	302 (229–339)
Caloric intake (kcal/day)	2058 (1552–2316)	2050 (1635–2546)
Protein intake (g/day)	75.4 (70.8–103.9)	113.1 (91.1–141.9)*
Total fat intake (g/day)	72.3 (66.9–83.1)	83.8 (70.1–131.5)
Saturated fat intake (g/day)	30.0 (20.2–33.3)	37.2 (29.1–47.2)*
Carbohydrate intake (g/day)	237.9 (156.0–287.9)	166.3 (142.2–215.4)
Dietary fructose intake (g/day)	30.6 (23.1–48.9)	1.0 (0.8–1.5)*
Alcohol intake (U/day)	0.6 (0.1–1.7)	0.1 (0.0–0.3)*
Smoking (% yes)	0%	20%

Data are expressed as median (interquartile range). * p < .05 versus healthy controls, analyzed with a Mann-Whitney U test. Parts of this table have already been published [11].

### 3.2 Blood pressure and pulse wave velocity

**Fig. 1:**
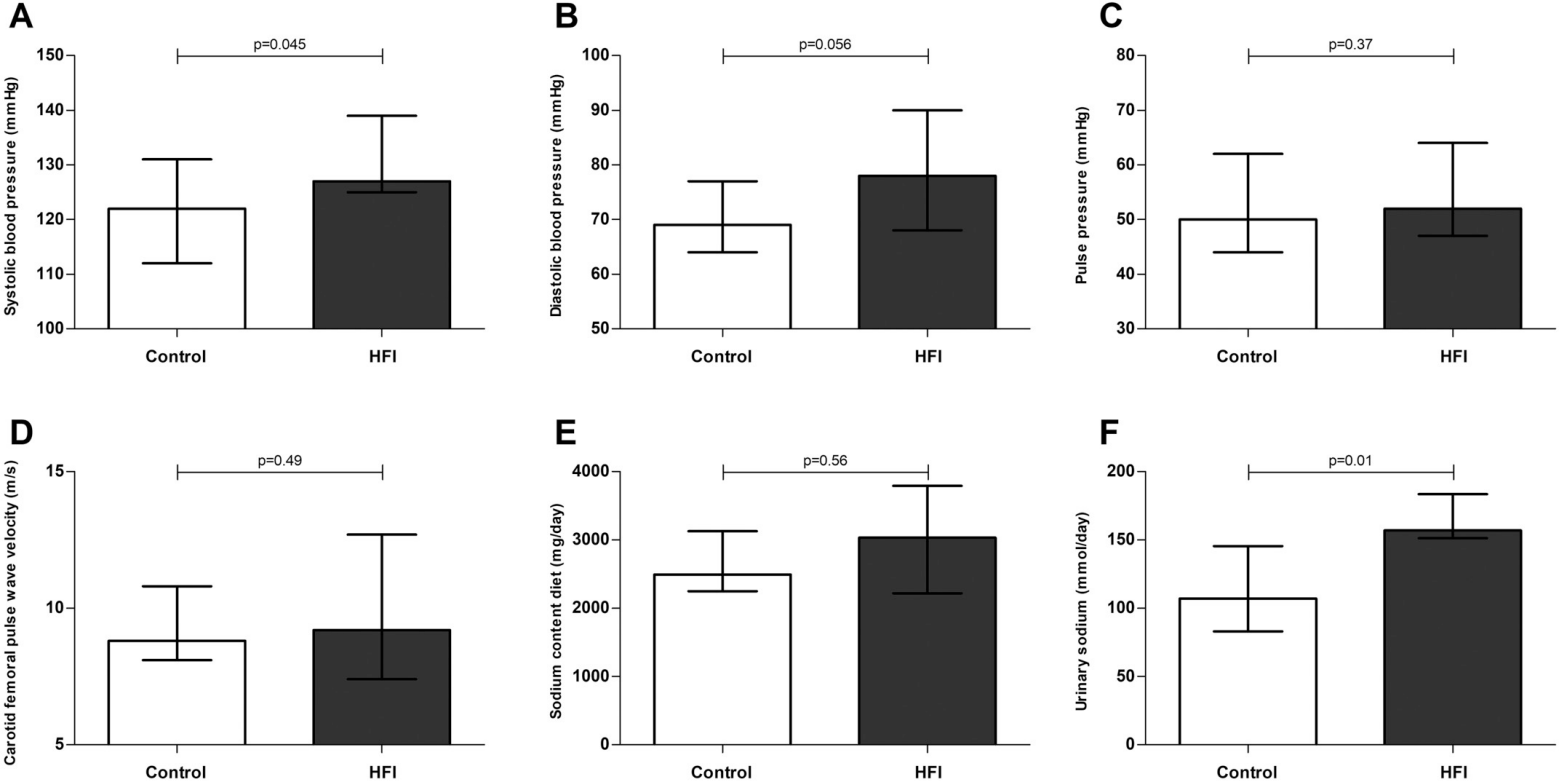
Blood pressure, aortic stiffness and sodium intake and excretion. Systolic blood pressure (panel A), diastolic blood pressure (panel B), pulse pressure (panel C), carotid-femoral pulse wave velocity (panel D), daily sodium intake (panel E) and 24-h urinary sodium excretion (panel F) in HFI patients (grey bars) and healthy controls (white bars). Data are presented as medians with interquartile range. Analyzed with a Mann-Whitney *U* test.

Both systolic and diastolic blood pressure were higher in HFI patients, of which only the former reached statistical significance (p = .045, Δ: 10 mmHg, 95%CI: 1;20, p = .056; Δ: 8 mmHg, 95%CI: -1;16; Fig. 1, Panel A and B, respectively). Five HFI patients had a systolic blood pressure ≥ 140 mmHg and/or diastolic blood pressure ≥ 90 mmHg, as opposed to one healthy control individual. Heart rate was not statistically different between HFI patients and healthy controls (*p* = .54, Δ: 2 bpm, 95%CI: -5;12). Pulse pressure and cf-PWV were not different either (*p* = .37, Δ: 2 mmHg, 95%CI: −4;11; and *p* = .49, Δ: 0.5 m/s, 95%CI: −1.4;2.9; Fig. 1, panel C and D, respectively). Dietary intake of sodium was not different between HFI patients and healthy controls (*p* = .56, Δ: 437 mg, 95%CI: −270;880; Fig. 1, Panel E). In contrast, urinary sodium excretion was higher in HFI patients (*p* = .01, Δ: 47.1 mmol/day, 95%CI: 9.1;81.1; Fig. 1, Panel F). Linear regression analyses were conducted to explore the role of smoking, cf-PWV, dietary sodium intake or urinary sodium excretion in the observed difference in systolic blood pressure. None of these had a substantial effect on the observed difference between HFI patients and controls (reduction in unstandardized beta of the affected state [HFI yes/no] after addition of the potential mediator: < 25%, data not shown).

### 3.3 Endothelial function

**Fig. 2:**
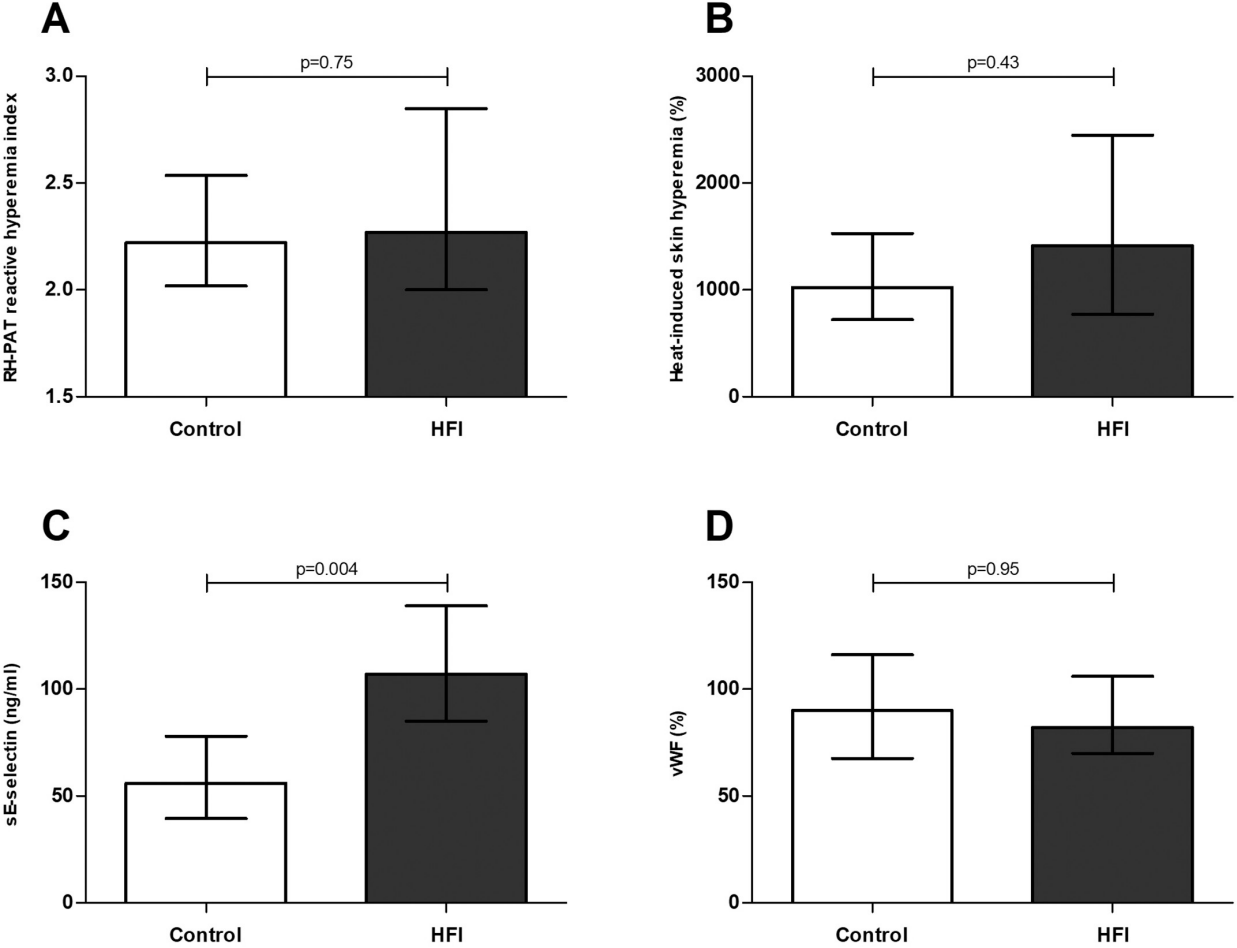
Endothelial function. Ischemia induced reactive hyperemia (panel A), heat-induced skin hyperemia (panel B), plasma sE-selectin levels (panel C) and plasma von Willebrand factor levels (panel D) in HFI patients (grey bars) and healthy controls (white bars). Data are presented as medians with interquartile range. Analyzed with a Mann-Whitney *U* test.

The reactive hyperemia index and percentage heat-induced skin hyperemia were not significantly different between HFI patients and healthy controls (*p* = .75, Δ: 0.09, 95%CI: −0.31;0.53; *p* = .43, Δ: 316%, 95%CI: −349;992; Fig. 2, Panel A and B, respectively). Plasma sE-selectin was significantly higher in HFI patients as compared to healthy controls (*p* = .004, Δ: 46 ng/ml, 95%CI: 19;71; Fig. 2, Panel C), whereas no significant difference was found for plasma vWF (*p* = .95, Δ: 1.5%, 95%CI: -19;20; Fig. 2, Panel D). Linear regression analyses were conducted to explore the role of plasma glucose, serum lipids, liver fat, alcohol intake, blood pressure, smoking behavior and eGFR_cr-cys_ in the observed difference in plasma sE-selectin. None of these caused a substantial reduction in the observed difference between HFI patients and controls.

### 3.4 Glomerular and proximal tubular function

**Fig. 3:**
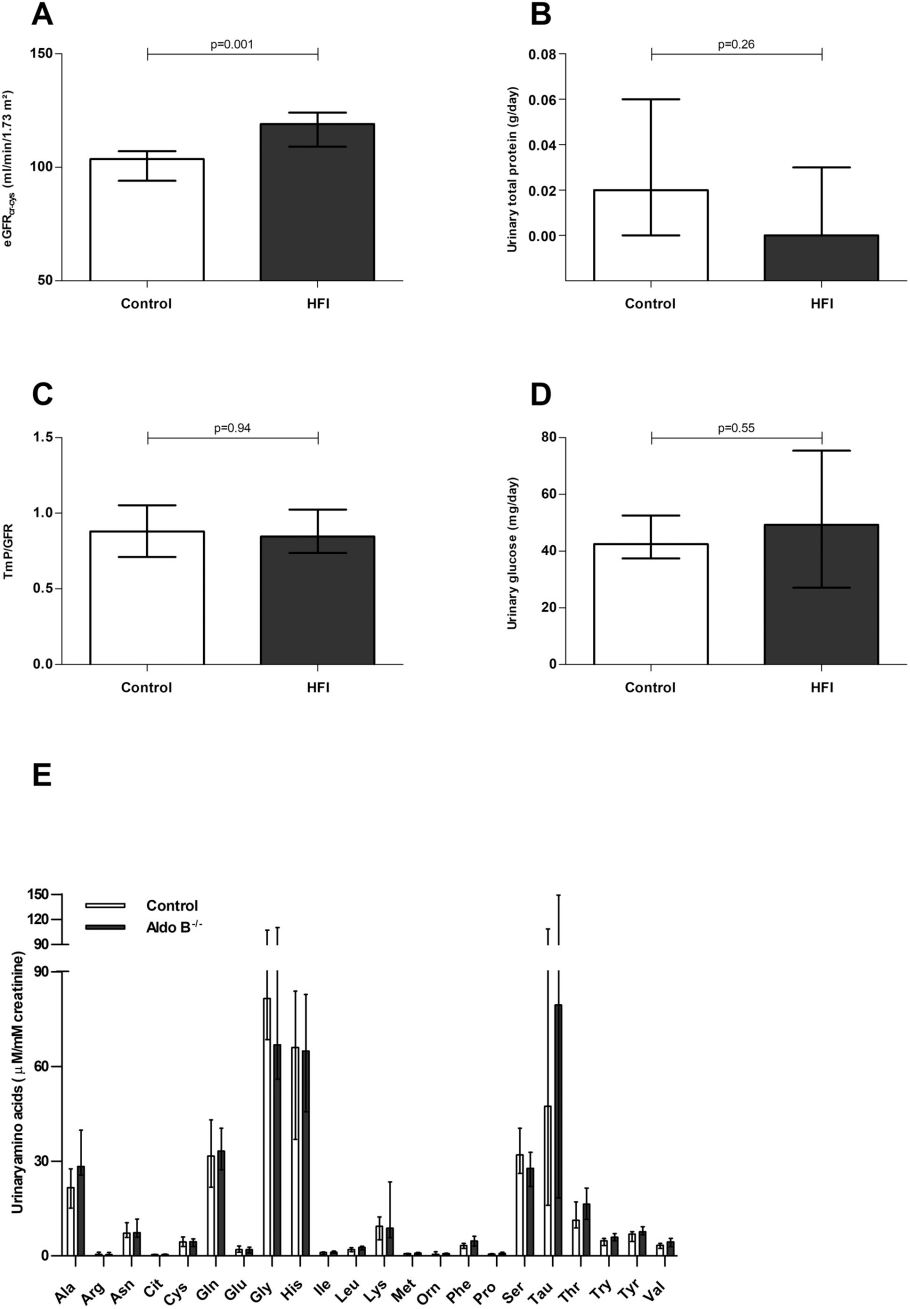
Kidney function. The eGFR_cr-cys_ (panel A) and 24-h urinary protein excretion (panel B), TmP/GFR (panel C), 24-h urinary glucose excretion (panel D) and 24-h urinary amino acid excretion (panel E) in HFI patients (grey bars) and healthy controls (white bars). Data are presented as medians with interquartile range. Analyzed with a Mann-Whitney *U* test. Abbreviations: Alanine (Ala), Arginine (Arg), Aasparagine (Asn), Citrulline (Cit), Cysteine (Cys), Glutamine (Gln), Glutamic acid (Glu), Glycine (Gly), Histidine (His), Isoleucine (Ile), Leucine (Leu), Lysine (Lys), Methionine (Met), Ornithine (Orn), Phenylalanine (Phe), Proline (Pro), Serine (Ser), Taurine (Tau), Threonine (Thr), Tryptophan (Try), Tyrosine (Tyr), Valine (Val)

The eGFR_cr_ and eGFR_cr-cys_ were significantly higher in HFI patients as compared to healthy controls (*p* < .001, Δ: 19 ml/min/1.73m^2^, 95%CI: 10;29; and *p* = .001, Δ: 16 ml/min/1.73m^2^, 95%CI: 6;24, respectively; Fig. 3, panel A). Although the estimate of difference for eGFR_cys_ was in a similar range (Δ 11 ml/min/1.73m^2^, 95%CI: −2;20), it did not reach statistical significance (*p* = .10). None of the participants had an eGFR <60 ml/min/1.73m^2^ (compatible with chronic kidney disease stage 3a). Linear regression analyses were conducted to explore the role of systolic blood pressure and dietary protein intake, which are known risk factors for glomerular hyperfiltration [30,31], in the observed difference in eGFR_cr-cys_. The unstandardized beta of the HFI affected state did not substantially decline after addition of these variables to the model (data not shown). Urinary total protein concentration was not statistically different between both groups (*p* = .26, Δ: 0.0 g/day, 95%CI:-0.03;0.0; Fig. 3, Panel B).

TmP/GFR and urinary glucose were not statistically different between both groups, as shown in Fig. 3, panel C and D (*p* = .94, Δ: 0.005, 95%CI: −0.22;0.18 and *p* = .55; Δ: 5.9 mg/day, 95%CI: −12.4;21.8, respectively). No statistical differences in urinary amino acid concentrations were found between HFI patients and healthy controls (Fig. 3, panel E). Alanine (Ala), valine (Val), leucine (Leu) and phenylalanine (Phe) concentrations tended to be higher in HFI patients (*p* = .06, Δ: 7.7 μM/mM creatinine, 95%CI: −1.1;17.4, *p* = .08, Δ: 1.2 μM/mM creatinine, 95%CI: −0.2;2.5, *p* = .10, Δ: 0.6 μM/mM creatinine, 95%CI: −0.07;1.2, and p = .08, Δ: 1.4 μM/mM creatinine, 95%CI: −0.06;2.7, respectively).

## 4 Discussion

HFI is an inborn error of metabolism that can be treated with a fructose-restricted diet. Up to now, mostly case reports on HFI at the time of diagnosis have been published [[32], [33], [34]]. We and others previously showed that HFI patients treated with a fructose-restricted diet are characterized by a greater intrahepatic triglyceride content [[11], [12], [13]]. In the present study, we investigated the long-term consequences of HFI on other aldolase-B-expressing organs and found that HFI patients have a higher systolic blood pressure, eGFR and sE-selectin levels compared to healthy controls matched for age, sex and BMI.

HFI patients displayed a higher systolic blood pressure than healthy controls. Diastolic blood pressure tended to be higher as well (*p* = .056). Five of 15 patients had systolic blood pressure ≥ 140 mmHg and/or diastolic blood pressure ≥ 90 mmHg. Since blood pressure was determined only twice during the study visit, we cannot exclude white coat hypertension as a potential explanation for the observed difference. Ideally, this would require 24-h blood pressure monitoring [35]. To explore the contribution of salt intake, which affects both systolic *and* diastolic blood pressure as an alternative explanation [36], we determined dietary sodium intake and 24-h urinary sodium excretion. Although the former was not statistically different between both groups, we did find a significantly higher 24-h urinary sodium excretion in HFI patients. Differences between intake and urinary excretion of sodium have been reported in other studies as well [37,38] and is most likely the result of inaccurate food recording. The National Academy of Medicine (formerly known as the Institute of Medicine) considers analysis of 24-h urine collection a reliable method of sodium intake estimation [39]. Regression analyses, however, did not show a substantial reduction in the unstandardized beta after addition of these variables to the model. Although these analyses suggest that dietary salt intake is not a major explanatory variable for the observed difference in systolic blood pressure, these exploratory analyses should be interpreted with caution given the relatively small study population under investigation. Similar conclusions can be drawn for the potential mediating role of aortic stiffness (approximated by cf-PWV) in explaining the higher systolic blood pressure in HFI. Regardless of the exact cause, it is clinically advisable to perform routine blood pressure measurements in HFI patients during outpatient visits.

Previous studies have shown that ingestion of fructose by HFI patients leads to proximal tubular dysfunction (i.e. Fanconi syndrome) and eventually to renal failure [9,40]. In the present study, proximal tubular function, reflected by TmP/GFR, urinary glucose and amino acid excretion, did not differ significantly between adult HFI patients treated with a fructose-restricted diet and healthy controls. Although the urinary alanine, valine, leucine and phenylalanine concentrations tended to be higher in HFI patients, all were within the normal range in comparison to the amino acid excretion as seen in patients with Fanconi syndrome [41]. In contrast, the eGFR_cr-cys_ was significantly higher in HFI patients than healthy controls. It is unlikely that this difference is explained by a difference in body (muscle) mass or diet – which affect serum creatinine levels [42,43] – since a similar effect size was observed for eGFR_cys_ (albeit not statistically significant, *p* = .10).

A higher eGFR could reflect glomerular hyperfiltration, as seen in other disease states such as diabetes mellitus [44], which could be a precursor of chronic kidney disease [45]. In the general population, a high GFR has been associated with an increase in albumin-to-creatinine ratio and incident albuminuria [46]. Of interest, both dietary protein intake and systolic blood pressure (which were found to be higher in HFI patients) are associated with glomerular hyperfiltration [30,31]. In addition, we previously showed that HFI patients are more glucose intolerant [11], which might contribute to glomerular hyperfiltration as well [47]. Regression analyses with systolic blood pressure and dietary protein intake as potential mediators did, however, not reveal a substantial reduction in the unstandardized beta. Again, a mediating role of these factors in explaining the higher eGFR in HFI patients cannot fully be excluded.

On the other hand, a higher eGFR in HFI might also reflect a truly better glomerular function. Although speculative, a hepatorenal axis in HFI patients may account for the current observations. We and others previously showed that HFI patients are also characterized by greater intrahepatic triglyceride content than controls [[11], [12], [13]]. Detailed studies in aldolase B deficient mice suggest that an increased dissociation of hepatic glucokinase from glucokinase regulatory protein (GKRP) may explain the greater intrahepatic triglyceride content in HFI [48]. Of interest, variants in the GKRP gene (that encode a GKRP protein that binds glucokinase less effectively [49]) have been associated with both an increased intrahepatic triglyceride content *and* a higher eGFR in the general population [50,51]. The same gene variants tended to protect from chronic kidney disease as well (*p* = .13) [51]. Moreover, liver-specific knockout of glucokinase results in increased kidney damage [52]. These suggestions of a hepatorenal axis in HFI deserve further study.

Of all measures of endothelial function, only plasma sE-selectin levels, an endothelium-specific biomarker, were significantly higher in HFI patients compared to healthy controls. The fact that plasma vWF, LDF and RH-PAT were not statistically different between HFI patients and healthy controls, might be due to a lack of statistical power, or alternatively, a difference in the type of blood vessel under investigation, i.e. small arteries (RH-PAT) versus skin arterioles (LDF) versus the microcirculation in general (plasma sE-selectin and vWF). Of note, previous epidemiological studies have shown that sE-selectin levels predict future type 2 diabetes and cardiovascular disease [53,54]. We previously showed that adult HFI patients treated with a fructose-restricted diet are more glucose intolerant than healthy controls [11].

This study has strengths and limitations. As hitherto mentioned, the small patient population makes it difficult to draw conclusions on non-significant results. Therefore, these results should be interpreted with caution. In addition, formal mediation analyses are required to explore the contribution of potential mediators to the difference in primary outcomes measures between HFI patients and healthy controls. Due to the low number of study participants, however, this was not possible. Last, the healthy control subjects, who were matched for age, sex and BMI, do not necessarily represent the general population.

This is the first report of HFI patients treated with a fructose-restricted diet using state of the art methods. Since only case reports on HFI at time of diagnosis have been published [[32], [33], [34]], this study provides useful information for the long-term management of HFI patients.

In conclusion, following our previous report on the presence of fatty liver, liver fibrosis and glucose intolerance [11], we now show that the adult HFI patients treated with a fructose-restricted diet are characterized by a higher systolic blood pressure, eGFR and soluble *E*-selectin level compared to age-, sex- and BMI-matched healthy controls. Further studies are required to elucidate the exact causes of these observations, either the direct consequence of the genetic defect or secondary to its dietary treatment. These results do emphasize the need for long-term follow-up of HFI patients, in particular with regard to blood pressure and kidney function.

## Author contribution statement

N.S. performed the measurements, conducted the analyses, researched the data and wrote the manuscript. A.J.H.M.H. conducted the LDF analyses, reviewed the manuscript and provided revisions to the manuscript. J.B. conducted the urinary amino acid analyses, reviewed the manuscript and provided revisions to the manuscript. E.J.F., J.A.P.B. and C.G.S. contributed to the measurements, reviewed the manuscript and provided revisions to the manuscript. C.E.M.H., F.G.D. and D.C. contributed to the inclusion of study participants, reviewed the manuscript and provided revisions to the manuscript. N.C.S., C.D.A.S., F.G.D., D.C. and M.C.G.J.B. conceived the study, supervised the analyses, researched the data, reviewed the manuscript and provided substantial revisions to the manuscript. M.C.G.J.B. is the guarantor of this work and, as such, had full access to all the data in the study and takes responsibility for the integrity of the data and the accuracy of the data analysis.

## Data availability

The datasets generated and/or analyzed during the current study are available from the corresponding author on reasonable request.

## References

[1] Ali M., Rellos P., Cox T.M. (1998). Hereditary fructose intolerance. J. Med. Genet..

[2] Cross N.C., de Franchis R., Sebastio G., Dazzo C., Tolan D.R., Gregori C., Odievre M., Vidailhet M., Romano V., Mascali G. (1990). Molecular analysis of aldolase B genes in hereditary fructose intolerance. Lancet (London, England).

[3] Fagerberg L., Hallstrom B.M., Oksvold P., Kampf C., Djureinovic D., Odeberg J., Habuka M., Tahmasebpoor S., Danielsson A., Edlund K., Asplund A., Sjostedt E., Lundberg E., Szigyarto C.A., Skogs M., Takanen J.O., Berling H., Tegel H., Mulder J., Nilsson P., Schwenk J.M., Lindskog C., Danielsson F., Mardinoglu A., Sivertsson A., von Feilitzen K., Forsberg M., Zwahlen M., Olsson I., Navani S., Huss M., Nielsen J., Ponten F., Uhlen M. (2014). Analysis of the human tissue-specific expression by genome-wide integration of transcriptomics and antibody-based proteomics. Mol. Cell. Proteomics.

[4] Zubay G. (1986). Biochemistry.

[5] Odievre M., Gentil C., Gautier M., Alagille D. (1978). Hereditary fructose intolerance in childhood. Diagnosis, management, and course in 55 patients. Am. J. Dis. Child..

[6] Baerlocher K., Gitzelmann R., Steinmann B., Gitzelmann-Cumarasamy N. (1978). Hereditary fructose intolerance in early childhood: a major diagnostic challenge. Survey of 20 symptomatic cases. Helv. Paediatr. Acta.

[7] Mock D.M., Perman J.A., Thaler M., Morris R.C. (1983). Chronic fructose intoxication after infancy in children with hereditary fructose intolerance. A cause of growth retardation. N. Engl. J. Med..

[8] von Ruecker A., Endres W., Shin Y.S., Butenandt I., Steinmann B., Gitzelmann R. (1981). A case of fatal hereditary fructose intolerance. Misleading information of formula composition. Helv. Paediatr. Acta.

[9] Levin B., Snodgrass G.J., Oberholzer V.G., Burgess E.A., Dobbs R.H. (1968). Fructosaemia. Observations on seven cases. Am. J. Med..

[10] Fernandes J., Saudubray J., Berghe G. Vd, Walter J. (2006). Inborn Metabolic Diseases.

[11] Simons N., Debray F.G., Schaper N.C., Kooi M.E., Feskens E.J.M., Hollak C.E.M., Lindeboom L., Koek G.H., Bons J.A.P., Lefeber D.J., Hodson L., Schalkwijk C.G., Stehouwer C.D.A., Cassiman D., Brouwers M. (2019). Patients with aldolase B deficiency are characterized by increased intrahepatic triglyceride content. J. Clin. Endocrinol. Metab..

[12] Aldamiz-Echevarria L., de Las Heras J., Couce M.L., Alcalde C., Vitoria I., Bueno M., Blasco-Alonso J., Concepcion Garcia M., Ruiz M., Suarez R., Andrade F., Villate O. (2020). Non-alcoholic fatty liver in hereditary fructose intolerance. Clin. Nutr..

[13] Di Dato F., Spadarella S., Puoti M.G., Caprio M.G., Pagliardini S., Zuppaldi C., Vallone G., Fecarotta S., Esposito G., Iorio R., Parenti G., Spagnuolo M.I. (2019). Daily fructose traces intake and liver injury in children with hereditary fructose intolerance. Nutrients.

[14] Liu J., Mak T.C., Banigesh A., Desai K., Wang R., Wu L. (2012). Aldolase B knockdown prevents high glucose-induced methylglyoxal overproduction and cellular dysfunction in endothelial cells. PLoS One.

[15] Cao W., Chang T., Li X.Q., Wang R., Wu L. (2017). Dual effects of fructose on ChREBP and FoxO1/3alpha are responsible for AldoB up-regulation and vascular remodelling. Clin. Sci..

[16] Liu J., Wang R., Desai K., Wu L. (2011). Upregulation of aldolase B and overproduction of methylglyoxal in vascular tissues from rats with metabolic syndrome. Cardiovasc. Res..

[17] World Medical Association Declaration of Helsinki (2013). Ethical principles for medical research involving human subjects. JAMA.

[18] Stehouwer C.D., Henry R.M., Ferreira I. (2008). Arterial stiffness in diabetes and the metabolic syndrome: a pathway to cardiovascular disease. Diabetologia.

[19] Laurent S., Cockcroft J., Van Bortel L., Boutouyrie P., Giannattasio C., Hayoz D., Pannier B., Vlachopoulos C., Wilkinson I., Struijker-Boudier H. (2006). A European network for non-invasive investigation of large, Expert consensus document on arterial stiffness: methodological issues and clinical applications. Eur. Heart J..

[20] Stehouwer C.D.A. (2018). Microvascular dysfunction and hyperglycemia: a vicious cycle with widespread consequences. Diabetes.

[21] Bonetti P.O., Pumper G.M., Higano S.T., Holmes D.R., Kuvin J.T., Lerman A. (2004). Noninvasive identification of patients with early coronary atherosclerosis by assessment of digital reactive hyperemia. J. Am. Coll. Cardiol..

[22] Rajan V., Varghese B., van Leeuwen T.G., Steenbergen W. (2009). Review of methodological developments in laser doppler flowmetry. Lasers Med. Sci..

[23] Sorensen B.M., Houben A.J., Berendschot T.T., Schouten J.S., Kroon A.A., van der Kallen C.J., Henry R.M., Koster A., Sep S.J., Dagnelie P.C., Schaper N.C., Schram M.T., Stehouwer C.D. (2016). Prediabetes and Type 2 diabetes are associated with generalized microvascular dysfunction: The Maastricht Study. Circulation.

[24] Choi P.J., Brunt V.E., Fujii N., Minson C.T. (1985). New approach to measure cutaneous microvascular function: an improved test of NO-mediated vasodilation by thermal hyperemia. J. Appl. Physiol..

[25] Levey A.S., Stevens L.A., Schmid C.H., Zhang Y.L., Castro A.F., Feldman H.I., Kusek J.W., Eggers P., Van Lente F., Greene T., Coresh J. (2009). A new equation to estimate glomerular filtration rate. Ann. Intern. Med..

[26] Waterval W.A., Scheijen J.L., Ortmans-Ploemen M.M., Habets-van der Poel C.D., Bierau J. (2009). Quantitative UPLC-MS/MS analysis of underivatised amino acids in body fluids is a reliable tool for the diagnosis and follow-up of patients with inborn errors of metabolism. Clin. Chim. Acta.

[27] Payne R.B. (1998). Renal tubular reabsorption of phosphate (TmP/GFR): indications and interpretation. Ann. Clin. Biochem..

[28] Rijksinstituut voor Volksgezondheid en Milieu (National Institute for Public Health and the Environment) (2010). NEVO-tabel (Dutch Food Composition Table). Nederlands Voedingsstoffenbestand 2010 (Dutch Food Composition Database 2010).

[29] Sluik D., Engelen A.I., Feskens E.J. (2015). Fructose consumption in the Netherlands: the Dutch National Food Consumption Survey 2007-2010. Eur. J. Clin. Nutr..

[30] Okada R., Yasuda Y., Tsushita K., Wakai K., Hamajima N., Matsuo S. (2012). Glomerular hyperfiltration in prediabetes and prehypertension. Nephrol. Dial. Transplant..

[31] Schwingshackl L., Hoffmann G. (2014). Comparison of high vs. normal/low protein diets on renal function in subjects without chronic kidney disease: a systematic review and meta-analysis. PLoS One.

[32] Chambers R.A., Pratt R.T. (1956). Idiosyncrasy to fructose. Lancet.

[33] Froesch E.R., Wolf H.P., Baitsch H., Prader A., Labhart A. (1963). Hereditary fructose intolerance. An inborn defect of hepatic fructose-1-phosphate splitting aldolase. Am. J. Med..

[34] Esposito G., Imperato M.R., Ieno L., Sorvillo R., Benigno V., Parenti G., Parini R., Vitagliano L., Zagari A., Salvatore F. (2010). Hereditary fructose intolerance: functional study of two novel ALDOB natural variants and characterization of a partial gene deletion. Hum. Mutat..

[35] Whelton P.K., Carey R.M., Aronow W.S., Casey D.E., Collins K.J., Dennison Himmelfarb C., DePalma S.M., Gidding S., Jamerson K.A., Jones D.W., MacLaughlin E.J., Muntner P., Ovbiagele B., Smith S.C., Spencer C.C., Stafford R.S., Taler S.J., Thomas R.J., Williams K.A., Williamson J.D., Wright J.T. (2018). 2017 ACC/AHA/AAPA/ABC/ACPM/AGS/APhA/ASH/ASPC/NMA/PCNA guideline for the prevention, detection, evaluation, and management of high blood pressure in adults: a report of the American College of Cardiology/American Heart Association task force on clinical practice guidelines. Hypertension.

[36] Sacks F.M., Svetkey L.P., Vollmer W.M., Appel L.J., Bray G.A., Harsha D., Obarzanek E., Conlin P.R., Miller E.R., Simons-Morton D.G., Karanja N., Lin P.H. (2001). Effects on blood pressure of reduced dietary sodium and the dietary approaches to stop hypertension (DASH) diet. DASH-Sodium Collaborative Research Group. N. Engl. J. Med..

[37] Kirkendall A.M., Connor W.E., Abboud F., Rastogi S.P., Anderson T.A., Fry M. (1976). The effect of dietary sodium chloride on blood pressure, body fluids, electrolytes, renal function, and serum lipids of normotensive man. J. Lab. Clin. Med..

[38] Schachter J., Harper P.H., Radin M.E., Caggiula A.W., McDonald R.H., Diven W.F. (1980). Comparison of sodium and potassium intake with excretion. Hypertension.

[39] Institute of Medicine (2010). Strategies to Reduce Sodium Intake in the United States.

[40] Lameire N., Mussche M., Baele G., Kint J., Ringoir S. (1978). Hereditary fructose intolerance: a difficult diagnosis in the adult. Am. J. Med..

[41] Norden A.G., Sharratt P., Cutillas P.R., Cramer R., Gardner S.C., Unwin R.J. (2004). Quantitative amino acid and proteomic analysis: very low excretion of polypeptides >750 Da in normal urine. Kidney Int..

[42] Vinge E., Lindergard B., Nilsson-Ehle P., Grubb A. (1999). Relationships among serum cystatin C, serum creatinine, lean tissue mass and glomerular filtration rate in healthy adults. Scand. J. Clin. Lab. Invest..

[43] Tangri N., Stevens L.A., Schmid C.H., Zhang Y.L., Beck G.J., Greene T., Coresh J., Levey A.S. (2011). Changes in dietary protein intake has no effect on serum cystatin C levels independent of the glomerular filtration rate. Kidney Int..

[44] Levine D.Z. (2008). Can rodent models of diabetic kidney disease clarify the significance of early hyperfiltration?: recognizing clinical and experimental uncertainties. Clin. Sci..

[45] Helal I., Fick-Brosnahan G.M., Reed-Gitomer B., Schrier R.W. (2012). Glomerular hyperfiltration: definitions, mechanisms and clinical implications nature reviews. Nephrology.

[46] Melsom T., Stefansson V., Schei J., Solbu M., Jenssen T., Wilsgaard T., Eriksen B.O. (2016). Association of increasing GFR with change in albuminuria in the general population. Clin. J. Am. Soc. Nephrol..

[47] Sun Z.J., Yang Y.C., Wu J.S., Wang M.C., Chang C.J., Lu F.H. (2016). Increased risk of glomerular hyperfiltration in subjects with impaired glucose tolerance and newly diagnosed diabetes. Nephrol. Dial. Transplant..

[48] Lanaspa M.A., Andres-Hernando A., Orlicky D.J., Cicerchi C., Jang C., Li N., Milagres T., Kuwabara M., Wempe M.F., Rabinowitz J.D., Johnson R.J., Tolan D.R. (2018). Ketohexokinase C blockade ameliorates fructose-induced metabolic dysfunction in fructose-sensitive mice. J. Clin. Invest..

[49] Beer N.L., Tribble N.D., McCulloch L.J., Roos C., Johnson P.R., Orho-Melander M., Gloyn A.L. (2009). The P446L variant in GCKR associated with fasting plasma glucose and triglyceride levels exerts its effect through increased glucokinase activity in liver. Hum. Mol. Genet..

[50] Speliotes E.K., Yerges-Armstrong L.M., Wu J., Hernaez R., Kim L.J., Palmer C.D., Gudnason V., Eiriksdottir G., Garcia M.E., Launer L.J., Nalls M.A., Clark J.M., Mitchell B.D., Shuldiner A.R., Butler J.L., Tomas M., Hoffmann U., Hwang S.J., Massaro J.M., O'Donnell C.J., Sahani D.V., Salomaa V., Schadt E.E., Schwartz S.M., Siscovick D.S., Voight B.F., Carr J.J., Feitosa M.F., Harris T.B., Fox C.S., Smith A.V., Kao W.H., Hirschhorn J.N., Borecki I.B. (2011). Genome-wide association analysis identifies variants associated with nonalcoholic fatty liver disease that have distinct effects on metabolic traits. PLoS Genet..

[51] Simons P., Simons N., Stehouwer C.D.A., Schalkwijk C.G., Schaper N.C., Brouwers M. (2018). Association of common gene variants in glucokinase regulatory protein with cardiorenal disease: A SYSTEMATIC review and meta-analysis. PLoS One.

[52] Gu Y., Mao Y., Li H., Zhao S., Yang Y., Gao H., Yu J., Zhang X., Irwin D.M., Niu G., Tan H. (2011). Long-term renal changes in the liver-specific glucokinase knockout mouse: implications for renal disease in maturity-onset diabetes of the young 2. Transl. Res..

[53] Meigs J.B., Hu F.B., Rifai N., Manson J.E. (2004). Biomarkers of endothelial dysfunction and risk of type 2 diabetes mellitus. Jama.

[54] Hwang S.J., Ballantyne C.M., Sharrett A.R., Smith L.C., Davis C.E., Gotto A.M., Boerwinkle E. (1997). Circulating adhesion molecules VCAM-1, ICAM-1, and E-selectin in carotid atherosclerosis and incident coronary heart disease cases: the Atherosclerosis Risk In Communities (ARIC) study. Circulation.

